# Pilot study to evaluate the need and implementation of a multifaceted nurse-led antimicrobial stewardship intervention in residential aged care

**DOI:** 10.1093/jacamr/dlae016

**Published:** 2024-02-14

**Authors:** Natali Jokanovic, Sue J Lee, Terry Haines, Sarah N Hilmer, Yun-Hee Jeon, Laura Travis, Darshini Ayton, Eliza Watson, Tess Tsindos, Andrew J Stewardson, Rhonda L Stuart, Allen C Cheng, Trisha N Peel, Anton Y Peleg, START Trial Group, Anton Peleg, Terry Haines, Allen Cheng, Trisha Peel, Kathryn Holt, Sarah Hilmer, Yun-Hee Jeon, Andrew Stewardson, Rhonda Stuart, Sue J Lee, Daniel Wilson, James Trauer, Marilyn Cruickshank, Nicola De Maio, Natali Jokanovic, Janine Roney, Jessica Wisniewski

**Affiliations:** Department of Infectious Diseases, The Alfred Hospital and Central Clinical School, Monash University, Melbourne, Victoria, Australia; Department of Infectious Diseases, The Alfred Hospital and Central Clinical School, Monash University, Melbourne, Victoria, Australia; School of Primary and Allied Health Care, Faculty of Medicine, Nursing and Health Sciences, Monash University, Melbourne, Victoria, Australia; Departments of Clinical Pharmacology and Aged Care, Kolling Institute of Medical Research, Royal North Shore Hospital, Faculty of Medicine and Health, The University of Sydney, Sydney, New South Wales, Australia; Sydney Nursing School, Faculty of Medicine and Health, The University of Sydney, Sydney, New South Wales, Australia; Department of Infectious Diseases, The Alfred Hospital and Central Clinical School, Monash University, Melbourne, Victoria, Australia; Health and Social Care Unit, School of Public Health and Preventive Medicine, Monash University, Clayton Victoria, Australia; Department of Infectious Diseases, The Alfred Hospital and Central Clinical School, Monash University, Melbourne, Victoria, Australia; Health and Social Care Unit, School of Public Health and Preventive Medicine, Monash University, Clayton Victoria, Australia; Department of Infectious Diseases, The Alfred Hospital and Central Clinical School, Monash University, Melbourne, Victoria, Australia; Public Health and Infection Prevention, Monash Health, Monash Medical Centre, Clayton, Victoria, Australia; Monash Infectious Diseases, Monash Health, Monash Medical Centre, Clayton, Victoria, Australia; Department of Infectious Diseases, The Alfred Hospital and Central Clinical School, Monash University, Melbourne, Victoria, Australia; Department of Infectious Diseases, The Alfred Hospital and Central Clinical School, Monash University, Melbourne, Victoria, Australia; Monash Biomedicine Discovery Institute, Infection and Immunity Theme, Department of Microbiology, Monash University, Clayton, Victoria, Australia

## Abstract

**Objectives:**

To evaluate the need and feasibility of a nurse-led antimicrobial stewardship (AMS) programme in two Australian residential aged care homes (RACHs) to inform a stepped-wedged, cluster randomized controlled trial (SW-cRCT).

**Methods:**

A mixed-methods pilot study of a nurse-led AMS programme was performed in two RACHs in Victoria, Australia (July–December 2019). The AMS programme comprised education, infection assessment and management guidelines, and documentation to support appropriate antimicrobial use in urinary, lower respiratory and skin/soft tissue infections. The programme was implemented over three phases: (i) pre-implementation education and integration (1 month); (ii) implementation of the intervention (3 months); and (iii) post-intervention evaluation (1 month). Baseline RACH and resident data and weekly infection and antimicrobial usage were collected and analysed descriptively to evaluate the need for AMS strategies. Feedback on intervention resources and implementation barriers were identified from semi-structured interviews, an online staff questionnaire and researcher field notes.

**Results:**

Six key barriers to implementation of the intervention were identified and used to refine the intervention: aged care staffing and capacity; access to education; resistance to practice change; role of staff in AMS; leadership and ownership of the intervention at the RACH and organization level; and family expectations. A total of 61 antimicrobials were prescribed for 40 residents over the 3 month intervention. Overall, 48% of antibiotics did not meet minimum criteria for appropriate initiation (respiratory: 73%; urinary: 54%; skin/soft tissue: 0%).

**Conclusions:**

Several barriers and opportunities to improve implementation of AMS in RACHs were identified. Findings were used to inform a revised intervention to be evaluated in a larger SW-cRCT.

## Introduction

Antimicrobial resistance (AMR) is a significant global concern and increasing within residential aged care homes (RACHs).^1,2^ Driven by high rates of antibiotic use (up to 80% of residents),^3,4^ which is often inappropriate,^4^ and the potential for AMR transmission across healthcare settings,^2,5^ RACHs remain an important setting to target antimicrobial stewardship (AMS) initiatives. Over 2018–19, 37% of Australian RACH residents were transferred to the emergency department and 31% admitted to a public hospital at least once,^6^ increasing the risk of AMR transmission and placing both residents and hospitalized patients at risk of complex infections, longer hospital stays and mortality.

AMS within hospitals is well established, with demonstrable reductions in antibiotic use;^7^ however, its impact and key components within RACHs remains uncertain.^7,8^ Interventions are frequently multifaceted and multidisciplinary, incorporating education, guidelines, and audit and feedback.^9^ Several studies have explored the specific role of nursing staff in AMS and infection prevention, finding reductions in antimicrobial use with nurse-led AMS initiatives but a need for tailored resources to bridge knowledge gaps and support active participation in AMS programmes.^10–12^ RACHs are complex settings and present several challenges for successful implementation of AMS, including staffing mix, workload, organizational structure, onsite availability of physicians and pharmacists, and increasingly complex residents with multimorbidity and cognitive impairment.^13^ Recent randomized controlled trials (RCTs) have sought to investigate the impact of AMS on antibiotic use and AMR in RACHs;^14,15^ however, few have explored the barriers and facilitators of implementing AMS,^16^ particularly in Australian RACHs.^17,18^

This study aimed to evaluate the need and feasibility of a nurse-led AMS intervention programme in two Australian RACHs to inform a larger stepped-wedge, cluster RCT (SW-cRCT).^15^

## Methods

### Study design and setting

A mixed-methods pilot study was performed in two RACHs in regional and metropolitan Victoria, Australia. Both RACHs provided 24 h nursing care with GP support. The study was performed over three phases between July 2019 and December 2019: (i) pre-implementation (1 month education and intervention integration); (ii) implementation (3 months); and (iii) post-intervention feedback (1 month). This included the collection of resident, facility, infection and antimicrobial use data over the three phases to support the needs assessment for AMS interventions in RACHs.

### Intervention

Full intervention procedures are detailed in the published protocol.^15^ In brief, the nurse-led AMS intervention comprised education, RACH-specific guidelines, documentation forms and fact sheets to support appropriate antimicrobial prescribing for urinary tract infections (UTIs), lower respiratory tract infections (LRTIs) and skin and soft tissue infections (SSTIs). Education included face-to-face education, an online interactive workbook with equivalent information, and fact sheets targeting improved diagnosis and antimicrobial management of common infections. This aligned with the specific intervention procedures to commence during the intervention phase. RACH staff, GPs and pharmacists had access to all resources. Both RACHs had no pre-existing AMS programme in place prior to the study.

Education was provided by the study coordinator (research pharmacist) to RACH staff, including registered nurses (RNs), enrolled nurses (ENs), personal care attendants (PCAs) and clinical managers. Education completion was strongly encouraged for all relevant staff in either mode (face-to-face or self-directed via written/online material). Residents and families received monthly face-to-face education and a fact sheet.

RACH-specific guidelines were developed to support the initial assessment and antimicrobial management of UTIs, LRTIs and SSTIs, adapted from existing antibiotic initiation criteria,^19^ infection surveillance^20^ and prescribing guidelines.^21^ Assessment guidelines included minimum signs and symptoms of infection for antibiotic initiation, investigations and considerations for hospitalization. During the intervention period, RACH staff were advised to refer to the guidelines if they suspected a resident had signs or symptoms of infection prior to referral to the GP. Other resources including the management guidelines and resident fact sheets were intended to be used by all RACH staff, GPs and pharmacists, as required along the infection assessment and management pathway.

### Implementation

To support implementation, the nursing leadership team [clinical manager(s), general manager] were consulted prior and during the intervention to tailor implementation. The leadership team selected a ‘nurse champion’ responsible for education completion, distribution and placement of resources onsite, and staff compliance with intervention procedures. Both RACHs selected a clinical manager, an RN by background, to provide study leadership onsite. Up to four face-to-face 1 h and twice-weekly 15 min education sessions at nursing handover meetings were provided at each RACH. Clinical managers prioritized the attendance of regularly rostered RNs/ENs for 1 h education as they were perceived to have the greatest input in intervention procedures (attendance of total employed including casual workforce: 30% RNs/ENs, 30%–35% PCAs).

### Data collection

Baseline RACH, staff and resident data included occupancy, staffing mix and resident characteristics. All systemic antimicrobial use, infections and hospitalizations were collected from paper-based residents’ medical records. These data were collected to provide insight into baseline infection and antimicrobial rates as the short intervention period was insufficient to evaluate intervention effectiveness.

Post-intervention qualitative feedback included one-on-one semi-structured interviews and an online staff questionnaire covering an understanding of antibiotic appropriateness and resistance, and usability and usefulness of intervention resources. The questionnaire (available as Supplementary data at *JAC-AMR* Online) primarily comprised questions on the Likert scale (strongly disagree to strongly agree) and additional open-ended questions to obtain feedback on each of the intervention resources. Interviews were audio-recorded and performed by two qualitative researchers (E.W. and T.T.). Researcher field notes documented observations related to intervention delivery.

### Analysis

Transcribed interviews and field notes were coded and thematically analysed independently by two researchers (N.J. and L.T.) using inductive and deductive approaches to identify implementation barriers (NVivo, v20.3). Questionnaire data were summarized descriptively and considered alongside the initial themes to develop the final list of themes. Resident and antimicrobial data were summarized descriptively (Stata, v17.0).

### Ethics

Ethical approval was obtained from the Alfred Hospital Human Research Ethics Committee (HREC) (HREC/18/Alfred/591). Consent to obtain data from residents’ medication records was waived. Written informed consent was obtained for interviews. Consent was implied on completion of the online questionnaire.

## Results

### Resident and RACH characteristics

RACH-1 comprised 60 beds serviced by up to 70 staff (15 RNs/ENs, 55 PCAs), 1 clinical manager and 16 GPs. RACH-2 comprised 75 beds serviced by up to 80 staff (20 RNs/ENs, 60 PCAs), 2 clinical managers and 10 GPs.

A total of 135 residents were enrolled (Table 1). Residents were primarily female (71%), median 90 (IQR: 83–93) years of age, diagnosed with dementia (60%), required full assistance with activities of daily living (70%), and resided in their RACH for a median 31 (IQR: 10–59) months.

**Table 1.** Resident baseline characteristics

	Total	RACH-1	RACH-2
	*N* = 135	*N* = 62	*N* = 73
Age, years, median (IQR)	90.0 (83.0–93.0)	92.0 (88.0–95.0)	87.0 (81.0–92.0)
Female, *n* (%)	96 (71.1)	52 (83.9)	44 (60.3)
Weight, kg, median (IQR)^a^	62.4 (55.3–75.6)	62.0 (55.0–72.6)	65.6 (55.8–78.9)
Length of stay, months, median (IQR)	31.0 (10.0–58.5)	34.0 (14.3–67.3)	31.0 (9.0–50.0)
Health conditions, *n* (%)			
Diabetes	26 (19.3)	8 (12.9)	18 (24.7)
COPD	13 (9.6)	7 (11.3)	6 (8.2)
Dementia	81 (60.0)	33 (53.2)	48 (65.8)
Peripheral vascular disease	10 (7.4)	4 (6.5)	6 (8.2)
Immunocompromised	9 (6.7)	6 (9.7)	3 (4.1)
Chronic kidney disease	16 (11.9)	13 (21.0)	3 (4.1)
Chronic wound	15 (11.1)	8 (12.9)	7 (9.6)
Pressure ulcer	5/15 (33)	2/8 (25)	3/7 (43)
Mobility, *n* (%)			
No assistance	0	—	—
Supervision	37 (27.4)	19 (30.6)	18 (24.7)
Full assistance	94 (69.6)	42 (67.7)	52 (71.2)
Bed bound	2/131 (1.5)	0/61 (0.0)	2/70 (2.9)
Not reported	4 (3.0)	1 (1.6)	3 (4.1)
CL_CR_, mL/min, *n* (%)	86 (63.7)	32 (51.6)	54 (74.0)
>50	38 (44)	9 (28)	29 (54)
31–50	33 (38)	13 (41)	20 (37)
≤30	15 (17)	10 (31)	5 (9)
Indwelling urinary catheter, *n* (%)	5 (3.7)	1 (1.6)	4 (5.5)
Urethral catheter	3 (2.2)	1 (1.6)	2 (2.7)
Suprapubic catheter	2 (1.5)	0 (0.0)	2 (2.7)
Smoker, *n* (%)	4 (3.0)	0 (0.0)	4 (5.5)
Hospitalization in prior 3 months, *n* (%)	21 (15.6)	9 (14.5)	12 (16.4)
Antimicrobial allergy label, *n* (%)	31 (23.0)	21 (33.9)	10 (13.7)
Recent antimicrobial use indications, *n* (%)	45 (33.3)	27 (43.5)	18 (24.7)
Respiratory infection	17 (38)	13 (48)	4 (22)
SSTIs	8 (18)	3 (11)	5 (28)
Urinary infection	7 (16)	4 (15)	3 (17)
Other infections	16 (36)	9 (33)	7 (39)
Prophylaxis^b^	14 (31)	8 (30)	6 (33)
Unclear/not reported	3 (7)	2 (7)	1 (6)
Vaccinations, *n* (%)^c^			
Influenza vaccination, 2019	91 (70.0)	46 (78.0)	45 (63.4)
Pneumococcal vaccination in prior 5 years	2 (1.5)	1 (1.7)	1 (1.4)

^a^ *n* = 129 non-missing values.

^b^No indication reported (*n* = 9), UTI (*n* = 2), respiratory infection (*n* = 2), splenectomy (*n* = 1).

^c^ *n* = 7 recorded vaccinations were missing administration dates.

### Antimicrobial use

A total of 124 suspected infections resulted in 61 antimicrobial prescriptions across 40 residents over 3 months (Table S1). Up to 21% of residents were prescribed an antimicrobial on any given month (range: 2%–21%). Antimicrobials were prescribed primarily for the treatment of UTIs (*n* = 13; 21%), RTIs (*n* = 22; 36%) and SSTIs (*n* = 15; 25%). Half of antibiotic prescriptions (*n* = 23/48; 48%) were inappropriate when assessed against minimum criteria (respiratory: 73%; urinary: 54%; skin/soft tissue: 0%).

### Implementation

Post-intervention feedback was obtained from seven one-on-one interviews (two clinical managers, three RNs, one general manager, one resident) and online questionnaire (*n* = 22). The questionnaire was completed by four RNs, two ENs, 13 PCAs, two clinical managers and one GP.

Of the questionnaire respondents, over 85% reported the resources were moderately to extremely useful in supporting improved antibiotic use and at least 77% were moderately to extremely likely to use them again. Improvements focused on simplifying education for PCAs and reducing content within guidelines and forms.

Six key themes related to implementation barriers identified from researcher field notes, interviews and the questionnaire included: aged care staffing and capacity; education completion; resistance to practice change; staff roles in AMS; leadership and intervention ownership at the RACH and organization level; and family expectations (Table 2). Key improvements included strategies to increase education accessibility and completion (online, mandatory completion, staff renumeration), guideline integration into standard operating procedures, and increased engagement and capacity building of RACH and organization-level leadership targeting uptake, compliance and intervention ownership.

**Table 2.** Barriers to implementation of antimicrobial stewardship and illustrative quotes from aged care staff^a^ interviews (*n* = 6), questionnaire (*n* = 22) and researcher field notes

Themes	Subthemes	Summary from interviews, questionnaire and researcher field notes	Illustrative quotes from staff interviews
Aged care staffing and capacity	Aged care staffing shortages. High staff turnover. Reliance on casual staffing. Time management pressures. Lack of confidence to challenge requests for antimicrobials.	RNs reported they were time-poor, overwhelmed with paperwork and were often short-staffed, resulting in reduced capacity to undertake education, provide adequate supervision of PCAs and integrate new procedures into their workflow. Reliance on casual staffing who are unfamiliar with new and existing procedures at the home. Clinical managers reported time management was often an issue and staff need to prioritize completing existing care over new initiatives and additional paperwork. Lack of confidence of aged care staff communicating and challenging family members’ beliefs around antimicrobial use.	*‘I’m run off my feet with lots of other things.’—RN 3* *‘I guess time management…Resident stuff takes time. So some days it’s really busy and there’s days when it’s you know stable…I think the main thing is trying to make sure the resident is safe and I don’t know clinically they are looked after, because there’s other things to be spending than going through paperwork I guess’—Clinical Manager 1*
Completion of education	Insufficient time to complete education. Lack of awareness to complete education. Prioritization of RNs and ENs over PCAs. Mode of delivery. Lack of mandatory education requirement. Lack of reimbursement to complete education.	Pre-existing face-to-face education sessions preferentially target RNs and ENs, are not always mandatory and may not be paid if outside staff member’s rostered hours. Clinical managers prioritized RN/EN attendance over PCAs, limiting the opportunity for upskilling of PCAs in AMS. Frequent short staffing and the absence of RN cover minimized attendance. Absence of payment for attendance to intervention education for staff who are not regularly rostered and unable to attend during working hours. Staff questionnaire: up to 60% prefer face-to-face education over online and/or written material.	*‘Being the RN on the floor, unless they replace me, it’s really, really hard to go off the floor for any length of time…so to actually get up to some of the lectures is just about impossible for me.’—RN 3* *‘Yeah definitely if it’s an at work thing we would definitely do it but I’m not really sure… if they directed it as mandatory we could do it. I mean for me I wasn’t really told we have to do this but if I get told to then I would definitely go through with that.’—RN 1*
Resistance to change	Reliance on existing behaviours, knowledge and practices. Perception of lack of inappropriate antimicrobial use.	Aged care staff reported difficulty changing existing behaviours and practices towards infection assessment and testing. Difficulty changing long-standing procedures, e.g. dipstick urine analysis/full ward tests in the absence of additional signs and symptoms beyond behaviour change. Aged care staff reported insufficient time to refer to intervention guidelines and instead relied on pre-existing clinical knowledge. Perception among aged care staff (all levels) that antimicrobial use was not necessarily a problem at their RACH.	*‘[Reverting back to existing practices] It’s just, it’s what I’ve always known. You know, people are resistant to change and it’s hard to adapt. It’s not that I don’t think it’s a great tool, I think it absolutely is, it’s just different. It’s something different that I’m not used to.’—Clinical Manager 2* *‘In terms of the guidance of it, has been helpful in some ways but there’s always factors that will stop that from happening which includes staff will do a full ward test if they think that the resident’s confused and they will continue to send it to the pathology to get it tested and also getting GPs involved in it as well would be a hard thing…and locums as well…like we can’t stop them from starting antibiotics’—Clinical Manager 1* *‘I don’t have time to actually look at it [resources] and I just use my basic knowledge, really nursing skills. I didn’t really use these forms although I think it would be helpful for new nurses to look at so I still continue with what my nursing skills are.—Clinical Manager 1* *‘The knowledge is improving but I think it’s more habit. I think its years and years and years of habit.’—RN 3*
Role of staff in antimicrobial stewardship	Difference in perceptions of role of PCAs. Self-perceived lack of impact on prescribing by aged care staff.	Clinical managers and RNs felt role of PCAs was limited to identification of early symptoms, RNs as primary decision-makers in the assessment of suspected infections including testing and referral. Knowledge gap among PCAs prevents an active role within AMS. Staff survey: 62% (*n* = 8/13) of PCAs agreed to strongly agreed to having an important role in AMS at their RACH. Aged care staff did not perceive they were well positioned to influence antimicrobial prescribing decisions and it was the sole role of the GP. Aged care staff ambivalence over their individual role and impact on antimicrobial prescribing. Community pharmacists had a limited role outside of supply due to time constraints.	*‘The PCAs here aren’t involved in this stuff, they do ADLs and feed people and give them drinks and that’s about all that goes mostly from what I can tell in this facility.’—RN 3* *‘I don’t think they [PCAs] really understand sometimes why we do stuff. They don’t see the advantage of telling you someone was vomiting and it’s like, this was Friday, it’s like well why didn’t you tell me? ‘Oh it was only a little vomit’. Well you report and I will decide if I follow it up or not. Subsequently the woman went to hospital, they didn’t understand that I need to know so there’s a gap there.’—RN 3* *‘I mean definitely there are some people that are on antibiotics constantly that shouldn’t be on them, but that’s not our clinical judgement along with that, that is the doctor’s…at the end of the day I’m just a registered nurse, I don’t have much leeway.’—Clinical Manager 2* *‘It would probably be good as a ‘we’ve tried doing this’ kind of thing, we’ve tried implementing new ways of trying to improve infections and antibiotic use but in the end it’s really up to the doctors who prescribe these antibiotics and all that stuff.’—Clinical Manager 1*
Leadership and ownership at the RACH and organization level	Lack of integration into standard operating procedures. Duplicated procedures. Insufficient awareness of new procedures. Insufficient reinforcement to follow new procedures. Competing pressures.	Lack of integration of intervention procedures into standard operating procedures and duplicated infection policies at the organization level. Staff duplicated notes across intervention forms, progress notes and incident-reporting software. Insufficient awareness and reinforcement from designated onsite trial leaders/clinical managers to complete education and refer to intervention guidelines and forms. Competing pressures including concurrent changes in other procedures and staffing at the facilities taking priority over implementation of the intervention.	*‘If [Aged Care Provider] says that this is what we have to do then I guess that’s something that we need to implement…but because it’s just a trial I think I’m sort of just like mmm you know, there’s other changes that I’ve had to implement that are more important than this, so I haven’t really encouraged the use of it I guess. There’s too many changes that are happening at the moment that outweighs this.’—Clinical Manager 1* *‘I mean if we get told ‘we have to use this one’ then definitely we can. I think it’s just that not all of us were really aware about that…but yeah, I think more ongoing reinforcement that I’m using these [suspected infection] forms and all that would help us.’—RN 1* *‘At this point what we do is write in the progress notes anyway, so filling in one of these [suspected infection forms] is repeating what you’ve already written so you sort of do it, I don’t know, as a plus not necessarily for any particular reason it’s just something extra you are doing on top of what you’re already doing.’—RN 3*
Expectations from family	Pressure to prescribe antimicrobials.	Pressure to prescribe antimicrobials from family members in the absence of appropriate indications for use.	*‘Sometimes it’s hard because we have family members that are insistent on their Mum or Dad having antibiotics, even though there is not really any clinical indication that they need it, like maybe the full ward test was clear or they were asymptomatic, but they are still quite insistent because they believe that Mum’s coming down with a UTI or chest infection, so we have to advocate, just give them the information they need and get the GP on board as well.’—General Manager*

ADL, activity of daily living.

^a^Aged care staff: RNs and ENs, PCAs and clinical manager.

## Discussion

This study evaluated the need and feasibility of implementing a nurse-led AMS programme across two RACHs to inform a multifaceted AMS intervention for an SW-cRCT. Half of all antibiotics for UTIs, LRTIs and SSTIs did not meet minimum criteria for initiation, supporting the need for strategies to improve appropriate antimicrobial use. Overall, the AMS programme was well received; however, several opportunities to improve implementation were identified. Implementation themes included staff perceptions of their role within AMS, and workforce and workload challenges,^17,18,22^ highlighting the complexities of implementing quality improvement programmes within RACHs.

Up to almost a quarter of residents were prescribed an antimicrobial on any given month, with a high level of variability between 2% and 21%. This prevalence is higher than the prevalence reported from the 2019 Australian Aged care National Antimicrobial Prescribing Survey of 568 Australian RACHs, which found that 5.5% of residents were prescribed at least one systemic antimicrobial,^23^ and higher than that found in 3052 RACHs across 24 European countries between 2016 and 2017, with a mean prevalence of 5.8%.^24^ Our findings are, however, lower than a review of prescription data of 3459 Australian RACH residents between 2016 and 2019, which identified a mean 18.9% of residents received an antibiotic monthly.^25^ These differences in estimates may be due to the variability in data collection time periods (point and period prevalence) and potential seasonal variability in antimicrobial prescribing. Approximately half of antibiotics prescribed in this pilot did not meet minimum criteria, comparable to previous Australian audits,^26^ highlighting the need for ongoing AMS strategies to improve their appropriate use in UTIs, LRTIs and SSTIs.

Opportunities to improve implementation targeted education uptake and perceptions of RACH staff roles within AMS. PCAs comprise 70% of the Australian RACH workforce^27^ and are often the first to identify symptoms and provide information to residents and families. Despite this, clinical managers and RNs did not perceive a significant role for PCAs within AMS, citing key knowledge gaps, conflicting with PCAs’ self-perceived role and interest in improving their knowledge. This is consistent with previous Australian RACH staff interviews, identifying exclusion of PCAs from AMS education despite interest to improve antimicrobial use.^18^ Aged care staff did not perceive that their role extended to influencing prescribing decisions, expressing it was the GP’s role and a lack of confidence in challenging decision-making, consistent with previous Australian aged care surveys.^28^ Nursing staff have previously been identified as having a key role in the multidisciplinary AMS team to support reductions in antimicrobial use and improve clinical outcomes,^10–12^ and highlighting the importance of addressing barriers to successful implementation. Addressing these knowledge and confidence gaps requires inclusive education and strategies to support attendance including flexible modes, dedicated time and remuneration for completion, and consideration of staff turnover to ensure new and temporary staff are also provided with education opportunities.

Workforce and workflow challenges are consistent barriers to implementation of quality improvement programmes.^18,22^ Staff turnover, insufficient number of RNs and reliance on agency staff were identified as key barriers in this pilot and internationally.^18^ Aged care staff were uncertain how to prioritize new AMS procedures in the setting of competing pressures and organizational changes. Tailoring AMS workflow under the constraints of existing workforce challenges and prioritizing AMS in RACHs is necessary to drive acceptance and adherence.

This study had several strengths and limitations. The intervention was championed at each RACH by existing clinical managers to support implementation. Regional and metropolitan sites provided broader representation of implementation barriers. Ongoing feedback during the intervention was provided informally by staff and contributed to revisions that informed the final intervention and implementation plan for an SW-cRCT. High staff turnover and poor availability of staff (particularly PCAs) at the time of evaluation limited our sample size. The short intervention period did not allow for the intervention to be evaluated for effectiveness; however, the refined intervention will be evaluated in an SW-cRCT. The absence of a pharmacist or dedicated staff member to provide AMS education may limit generalizability and supports the need to expand modes of education delivery to improve accessibility and translation beyond this pilot. Although both RACHs were managed by the same provider, variability in culture, workforce and procedures may limit generalizability to other RACHs more broadly.

## Conclusions

This pilot identified a need for AMS strategies to improve the appropriateness of antibiotic use and several barriers and opportunities to improve its implementation in RACHs. Tailored strategies are required to improve education uptake and capacity of nursing staff to lead AMS programmes in the context of workforce and workflow challenges in RACHs. Findings were used to inform a revised intervention to be evaluated in an SW-cRCT across 12 RACHs.

## Supplementary Material

dlae016_Supplementary_Data

## Members of the START Trial Group

START trial Principal Investigator: Professor Anton Peleg. START co-investigators: Professor Terry Haines, Professor Allen Cheng, Professor Trisha Peel, Professor Kathryn Holt, Professor Sarah Hilmer, Professor Yun-Hee Jeon, Associate Professor Andrew Stewardson, Professor Rhonda Stuart. Statistician: Dr Sue J. Lee. Associate investigators: Dr Daniel Wilson, Associate Professor James Trauer, Professor Marilyn Cruickshank, Dr Nicola De Maio, Bupa Aged Care Australia, Bupa Health Foundation. START Trial Coordination and Administration: Dr Natali Jokanovic (START Clinical Trial Coordinator), Ms Janine Roney. Department of Infectious Diseases, Microbiology Laboratory: Dr Jessica Wisniewski.

## Supplementary data

Post-pilot questionnaire, Table S1 and Minimum antibiotic criteria are available as Supplementary data at *JAC-AMR* Online.
