# Supplementary material for: Pilot study to evaluate the need and implementation of a multifaceted nurse-led antimicrobial stewardship intervention in residential aged care
Source: JAC Antimicrob Resist. 2024 Feb 14;6(1):dlae016. doi: 10.1093/jacamr/dlae016 (PMC10873136; doi:10.1093/jacamr/dlae016)
Supplement: dlae016_Supplementary_Data [file dlae016_supplementary_data.docx]

**Supplementary data**

**Pilot study of a multifaceted nurse-led antimicrobial stewardship intervention in residential aged care.**

**Table S1.** Antimicrobials prescribed over the three-month intervention period, by indication (n=61 prescriptions from 40 residents).

|  | **Total** | **RACH-1** | **RACH-2** |
| --- | --- | --- | --- |
| Residents prescribed an antimicrobial, n | 40 | 16 | 24 |
| Antimicrobial prescriptions, n | **61** | **26** | **35** |
| **Indications, n (%)*** |  |  |  |
| **Urinary tract infections, n (%)** | **13 (21)** | **6 (23)** | **7 (20)** |
| UTI | 7 | 5 | 2 |
| Catheter-associated UTI | 6 | 1 | 5 |
| **Respiratory tract infection, n (%)** | **22 (36)** | **6 (23)** | **16 (46)** |
| Nonspecific respiratory infection | 13 | 4 | 9 |
| Pneumonia | 5 | 1 | 4 |
| Other | 4 | 1 | 3 |
| **Skin/Soft tissue infections, n (%)** | **15 (25)** | **6 (23)** | **9 (26)** |
| Nonspecific wound | 2 | 1 | 1 |
| Cellulitis | 2 | 2 | 0 |
| Ulcer | 5 | 2 | 3 |
| Shingles | 2 | 0 | 2 |
| Other | 4 | 1 | 3 |
| **Prophylaxis, n (%)** | **2 (3)** | **1 (4)** | **1 (3)** |
| Post-surgery | 1 | 1 | 0 |
| Urinary tract infection | 1 | 0 | 1 |
| **Other infections, n (%)**† | **5 (8)** | **4 (15)** | **1 (3)** |
| **Unclear/not reported, n (%)** | **4 (7)** | **3 (12)** | **1 (3)** |
| **Met minimum criteria for infection** |  |  |  |
| Yes | 25 (41) | 11 (42) | 14 (40) |
| No | 23 (38) | 7 (27) | 16 (46) |
| Not applicable^2^ | 13 (21) | 8 (31) | 5 (14) |

*Denominator is total number of antimicrobial prescriptions. Antimicrobials may have greater than one documented indication.

† Indications outside of urinary, respiratory and skin/soft tissue infections.

**Post-pilot questionnaire - Questions**

1. What is your profession?
   - Registered nurse
   - Enrolled nurse
   - Personal care attendant (PCA)/Assistant in Nursing (AIN)
   - General practitioner
   - Pharmacist
   - Other
2. Please rate how strongly you agree or disagree with the following statements

| Strongly disagree | | Disagree | Somewhat disagree | Neither agree nor disagree | Somewhat agree | Agree | Strongly agree |
| --- | --- | --- | --- | --- | --- | --- | --- |
| I have an important role in antimicrobial stewardship | o | o | o | o | o | o | o |
| I have a good understanding of antibiotic resistance | o | o | o | o | o | o | o |
| I feel confident to assess common infections | o | o | o | o | o | o | o |
| I feel confident to manage common infections | o | o | o | o | o | o | o |
| Antibiotics are used appropriately in my facility | o | o | o | o | o | o | o |
| I feel pressured to give antibiotics to residents | o | o | o | o | o | o | o |

The following questions relate to components of the START antimicrobial stewardship program that was piloted at your facility.

The program included the following resources:

- **Education:** face-to-face, online workbook, fact sheets
- **Guidelines**: assess and manage common infections
- **Documentation forms:** record assessment and management of infections
- **Resident/families fact sheet:** information on appropriate antibiotic use

The following infections were targeted:

- Urinary tract infections
- Lower respiratory tract infections
- Skin and soft tissue infections

1. Which START resources were you **aware** of? *(Select all that apply)*

▢ Education

▢ Guidelines

▢ Documentation forms

▢ Resident/family fact sheet

▢ None of the above

*Display This Question:*

*If Which START resources were you aware of? (Select all that apply) = Education*

3a. Did you attend an **education session or access the workbook?**

- - Yes
  - No

*Display This Question:*

*If Did you attend an education session or access the workbook? = Yes*

3ai. In regards to **education**, please rate how strongly you agree or disagree with the following statements

| Strongly disagree | | Disagree | Somewhat disagree | Neither agree nor disagree | Somewhat agree | Agree | Strongly agree |
| --- | --- | --- | --- | --- | --- | --- | --- |
| The content was relevant to my practice | o | o | o | o | o | o | o |
| There was too much content | o | o | o | o | o | o | o |
| The content was easy to understand | o | o | o | o | o | o | o |
| There was information I did not know before | o | o | o | o | o | o | o |
| I would like more education | o | o | o | o | o | o | o |
| Overall, I am satisfied with the education | o | o | o | o | o | o | o |

*Display This Question:*

*If Did you attend an education session or access the workbook? = No*

3aii. Which of the following reasons best describes why you **did not attend or access the education?**

- - I did not have time
  - It is too long or complicated
  - I have adequate knowledge of this topic
  - It is not relevant to my practice
  - Other

*Display This Question:*

*If Which START resources were you aware of? (Select all that apply) = Guidelines*

3b. Did you use any of the **guidelines?**

- - Yes
  - No

*Display This Question:*

*If Did you use any of the guidelines? = Yes*

3bi. In regards to **guidelines**, please rate how strongly you agree or disagree with the following statements

|  | Strongly disagree | Disagree | Somewhat disagree | Neither agree nor disagree | Somewhat agree | Agree | Strongly agree |
| --- | --- | --- | --- | --- | --- | --- | --- |
| The content is relevant to my practice | o | o | o | o | o | o | o |
| There is too much content | o | o | o | o | o | o | o |
| The content is easy to understand | o | o | o | o | o | o | o |
| I would like more training to use them | o | o | o | o | o | o | o |
| Overall, I am satisfied with the guidelines | o | o | o | o | o | o | o |

*Display This Question:*

*If Did you use any of the guidelines? = No*

3bii. Which of the following reasons best describes why you **did not use the guidelines?**

- - I did not have time to use them
  - They are too long or complicated
  - I have adequate knowledge of this topic
  - My resident(s) did not have an infection
  - They are not relevant to my practice
  - Other

*Display This Question:*

*If Which START resources were you aware of? (Select all that apply) = Documentation forms*

3c. Did you use any of the **documentation forms?**

- - Yes
  - No

*Display This Question:*

*If Did you use any of the documentation forms? = Yes*

3ci. In regards to **documentation forms**, please rate how strongly you agree or disagree with the following statements

| Strongly disagree | | Disagree | Somewhat disagree | Neither agree nor disagree | Somewhat agree | Agree | Strongly agree |
| --- | --- | --- | --- | --- | --- | --- | --- |
| They are relevant to my practice | o | o | o | o | o | o | o |
| There is too much content | o | o | o | o | o | o | o |
| They are easy to complete | o | o | o | o | o | o | o |
| I would like more training to use them | o | o | o | o | o | o | o |
| Overall, I am satisfied with the forms | o | o | o | o | o | o | o |

*Display This Question:*

*If Did you use any of the documentation forms? = No*

3cii. Which of the following reasons best describes why you **did not use the documentation forms?**

- - I did not have time to complete them
  - They are too long or complicated
  - I do not feel they are needed
  - My resident(s) did not have an infection
  - They are not relevant to my practice
  - Other

*Display This Question:*

*If Which START resources were you aware of? (Select all that apply) = Resident/family fact sheet*

3d. Did you use the **fact sheets for residents and families?**

- - Yes
  - No

*Display This Question:*

*If Did you use the fact sheets for residents and families? = Yes*

3di In regards to the **resident/family fact sheet**, please rate how strongly you agree or disagree with the following statements

|  | Strongly disagree | Disagree | Somewhat disagree | Neither agree nor disagree | Somewhat agree | Agree | Strongly agree |
| --- | --- | --- | --- | --- | --- | --- | --- |
| The content is relevant for residents/families | o | o | o | o | o | o | o |
| There is too much content | o | o | o | o | o | o | o |
| The content is easy to understand | o | o | o | o | o | o | o |
| Overall, I am satisfied with the fact sheet | o | o | o | o | o | o | o |

*Display This Question:*

*If Did you use the fact sheets for residents and families? = No*

3dii. Which of the following reasons best describes why you **did not use the fact sheet for residents/families?**

- - I did not have time to use them
  - It was too long or complicated
  - I do not find them useful
  - My resident and/or their family did not need one
  - Other

1. What is your preferred mode to receive education? *(Select all that apply)*

▢ Face-to-face individual session

▢ Face-to-face group session

▢ Online

▢ Fact sheets

▢ Other

1. How **useful** were the following START resources in improving how antibiotics were used?

*If you have not used a resource, indicate how useful you feel it would be to your practice.*

| Extremely useless | | Moderately useless | Slightly useless | Neither useful nor useless | Slightly useful | Moderately useful | Extremely useful |
| --- | --- | --- | --- | --- | --- | --- | --- |
| Education | o | o | o | o | o | o | o |
| Guidelines | o | o | o | o | o | o | o |
| Documentation forms | o | o | o | o | o | o | o |
| Resident/family fact sheet | o | o | o | o | o | o | o |

1. How likely are you to use the following START resources in future?

| Extremely unlikely | | Moderately unlikely | Slightly unlikely | Neither likely nor unlikely | Slightly likely | Moderately likely | Extremely likely |
| --- | --- | --- | --- | --- | --- | --- | --- |
| Education | o | o | o | o | o | o | o |
| Guidelines | o | o | o | o | o | o | o |
| Documentation forms | o | o | o | o | o | o | o |
| Resident/family fact sheet | o | o | o | o | o | o | o |

1. Do you have any comments or suggestions to improve the START resources?
2. Do you have any other feedback for the START project?

**Minimum antibiotic criteria for appropriateness**

**Urinary tract infections**

- If indwelling catheter:
  - At least one: temp ≥38C, acute change in mental status, flank/loin pain or tenderness, shaking/shivering
- If no indwelling catheter:
  - Painful urination
  - If no painful urination
    - Temp ≥38C or acute change in mental status

AND

- - - At least one new or worsening symptom:
      - Urge to urinate
      - Frequency of urination
      - Lower abdominal pain or tenderness
      - Visible blood in urine
      - Flank/loin pain or tenderness
      - Urinary incontinence

**Lower respiratory tract infections**

- If febrile (temp ≥38C):
  - New or increasing cough

AND

- - At least one: heart rate >100 bpm, shaking/shivering, acute change in mental status, respiratory rate > 25 breaths/min
- If not febrile (temp <38C):
  - If diagnosis of COPD present: new or increased cough and purulent sputum
  - If no diagnosis of COPD present: new or increased cough and purulent sputum and at least one: acute change in mental status, respiratory rate >25 breaths/min

**Skin and soft tissue infections**

- New or increasing purulent drainage at a wound, skin or soft-tissue site OR
- At least 2 new or increasing symptoms at the affected site: redness, tenderness, warmth, swelling, temp ≥38C
